# Antiproliferative Sorbicillinoids From the Deep-Sea-Derived *Penicillium allii-sativi*

**DOI:** 10.3389/fmicb.2020.636948

**Published:** 2021-01-21

**Authors:** Chun-Lan Xie, Duo Zhang, Ting Lin, Zhi-Hui He, Qing-Xiang Yan, Qi Cai, Xiao-Kun Zhang, Xian-Wen Yang, Hai-Feng Chen

**Affiliations:** ^1^ School of Pharmaceutical Sciences, Xiamen University, Xiamen, China; ^2^ Key Laboratory of Marine Biogenetic Resources, Third Institute of Oceanography, Ministry of Natural Resources, Xiamen, China

**Keywords:** deep-sea, fungi, sorbicillinoids, cell cycle, cytotoxicities

## Abstract

Two new (**1**–**2**) and three known (**3**–**5**) sorbicillinoids were isolated from the deep-sea-derived fungus *Penicillium allii-sativi* MCCC 3A00580. Compounds **1** and **2**, named sorbicatechols C and D, were two new hybrid dihydrosorbillinoids. Their structures were established mainly by spectroscopic analyses and electronic circular dichroism (ECD) calculations. All five isolates were tested for antiproliferative activities against four tumor cell lines of MCF-7, HT-29, HuH-7, and LNCap. Compounds **2** and **5** inhibited HT-29 cells in a good dose-dependent manner. Mechanism investigation uncovered that they could significantly induce cell cycle G2-M phase arresting by increasing the protein levels of p-H3 and cyclin B1.

## Introduction

**Figure 1:**
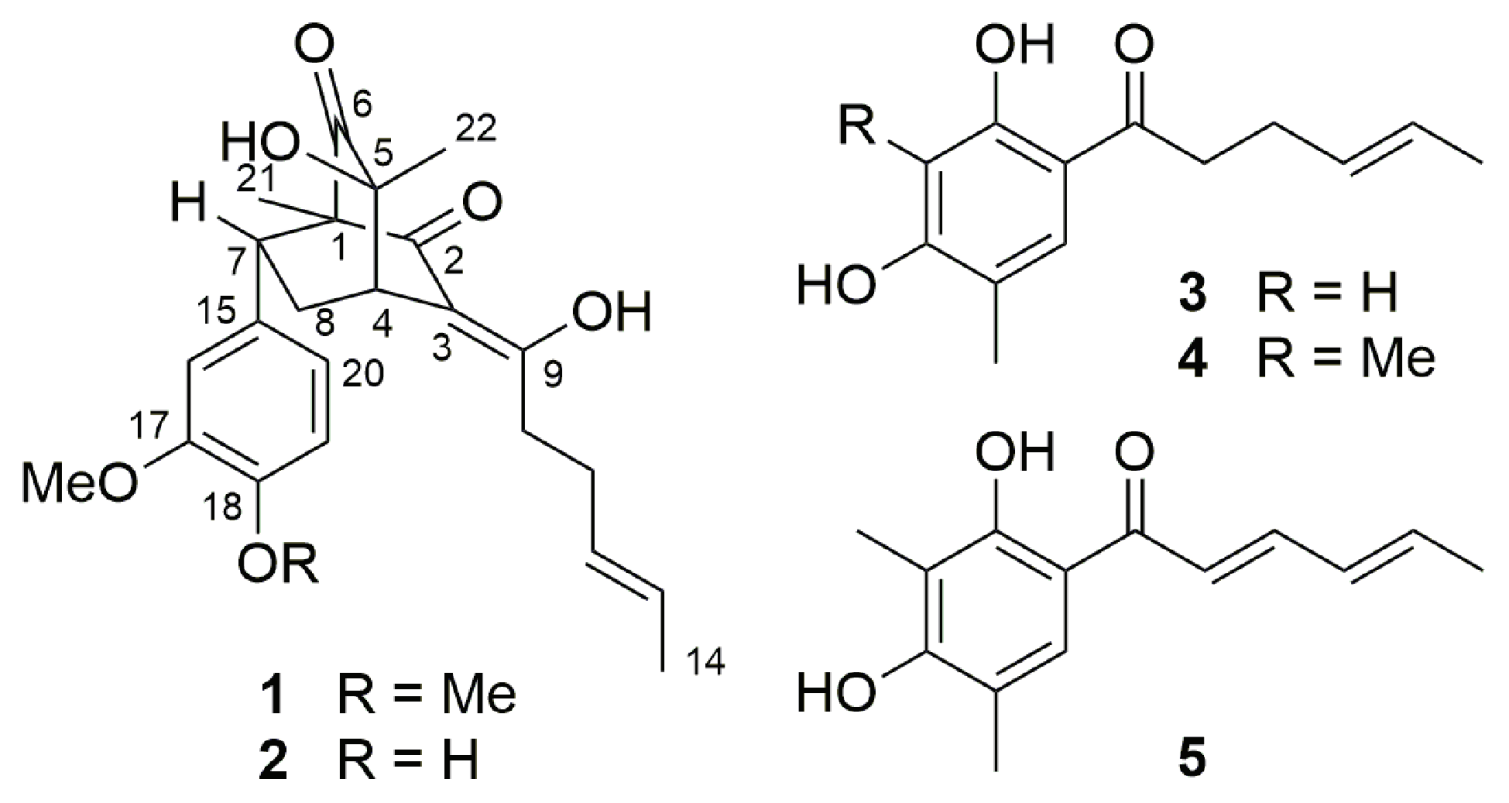
Compounds **1**–**5** from *Penicillium allii-sativi* MCCC 3A00580.

Sorbicillinoids are hexaketide metabolites that possess complex and highly oxygenated frameworks. Structurally, they can be divided into four groups: monomeric, dimeric, trimeric, and hybrid sorbicillinoids (Harned and Volp, 2011; Meng et al., 2016). The unique structural features of the sorbicillinoids make them attractive candidates for developing new pharmaceutical and agrochemical agents (Abe et al., 2000, 2001; Fahad et al., 2013; Meng et al., 2019; Kahlert et al., 2020). Up to now, around 100 sorbicillinoids have been reported from fungi, especially marine *Penicillium* (Meng et al., 2016). During our ongoing search for structurally novel and biologically interesting secondary metabolites from deep-sea-derived microorganisms (Yang et al., 2013; Niu et al., 2017, 2020; Xie et al., 2019a), the rice static fermentation extract of *Penicillium allii-sativi* MCCC 3A00580 exhibited potent *in vitro* antitumor activity. Previously, three meroterpenoids were obtained and andrastones A showed significant inhibitory effect against HepG2 tumor cells by activating caspase-3 and regulating the transcriptional activation function of RXR*α* (Xie et al., 2019b). Further investigation on this strain led to the discovery of two new and three known sorbicillinoid derivates (Figure 1). Structurally, compounds **1** and **2** are two novel hybrid dihydrosorbicillinoids. All isolates were tested for antiproliferative bioactivities, compounds **2** and **5** could inhibit HT-29 tumor cells in a good dose-dependent manner. Herein, we report the isolation, structures, and bioactivities of these compounds.

## Materials and Methods

### General Experimental Procedures

The NMR spectra were recorded on a Bruker 400 MHz spectrometer using TMS as an internal standard. The high resolution electrospray ionization mass spectroscopy (HRESIMS) spectra were measured on a Waters Xevo G2 Q-TOF (Waters) mass spectrometer. Optical rotations were measured with an Anton Paar MCP100 polarimeter. Electronic circular dichroism (ECD) spectra were measured on a JASCO J-715 spectropolarimeter. TLC and column chromatography (CC) were performed on plates precoated with silica gel GF254 (10–40 μm) or over silica gel (200–300 mesh, Qingdao Marine Chemical Factory). Chromatography was performed using Sephadex LH-20 (18–110 μm, Amersham Pharmacia Biotech AB), and octadecylsilane (ODS) silica gel (50 μm, Daiso). The preparative and semipreparative HPLC were performed on an Agilent Technologies 1,260 infinity instrument equipped with the DAD detector. Anti-*β*-actin (Cat. 4970S), anti-p-H3 (Cat. 3,377), and anti-Cyclin B1 (Cat. 12231S) antibodies were all purchased from Cell Signaling Technology (Boston, MA, United States).

### Fermentation, Extraction, and Isolation

*Penicillium allii-sativi* was isolated from the western Pacific Ocean (−4,302 m). The identification and fermentation of the fungus were reported previously (Xie et al., 2019b). As a result, a defatted extract (60.4 g) was obtained, which was subjected to CC over silica gel with gradient CH_2_Cl_2_-MeOH to get eight fractions (Fr.1–Fr.8). Fraction Fr.3 (5.5 g) was further purified by CC on ODS using a gradient H_2_O-MeOH to yield three subfractions (Fr.3.1–Fr.3.3). Compounds **1** (23.2 mg) and **2** (4.5 mg) were obtained from subfraction Fr.3.2 (211.4 mg) by repeated CC over silica gel CC (PE-EtOAc, 3:1) and Sephadex LH-20 (MeOH). While compound **3** (4.4 mg) was obtained from subfraction Fr.3.3 (150.6 mg) by CC over silica gel (CH_2_Cl_2_-MeOH, 50:1) and Sephadex LH-20 (MeOH). Fraction Fr.8 (15 g) was subjected subsequently to CC over ODS (MeOH-H_2_O: 5%→100%) and silica gel (CH_2_Cl_2_-MeOH, 30:1) to get compounds **4** (20.3 mg) and **5** (16.5 mg).

*Sorbicatechol C (**1**)*: yellow oil; [*α*]20D−14.6 (*c* 0.50, MeOH), −7.2 (*c* 0.50, CHCl_3_); UV (CH_3_OH) *λ*_max_ (log *ε*) 257 (3.24), 288 (3.61) nm; ECD (MeOH) *λ*_max_ (Δ*ε*) 201 (−4.63), 231 (+0.68), 297 (−3.29), 337 (+1.77) nm; ^1^H and ^13^C NMR data, see Table 1; HRESIMS *m/z* 437.1951 [M+Na]^+^ (calcd for C_24_H_30_O_6_Na, 437.1940), 413.1958 [M−H]^−^ (calcd for C_24_H_29_O_6_, 413.1964).
*Sorbicatechol D (**2**)*: yellow oil; [*α*]20 D −19.1 (*c* 0.35, MeOH); UV (Me) *λ*_max_ (log *ε*) 268 (2.42), 286 (2.50) nm; ECD (MeOH) *λ*_max_ (Δ*ε*) 230 (−0.87), 231 (+2.24), 296 (−12.30), 335 (+11.58) nm; ^1^H and ^13^C NMR data, see Table 1; HRESIMS *m/z* 399.1790 [M−H]^−^ (calcd for C_23_H_27_O_6_, 399.1808).

**Table 1:** ^1^H and ^13^C NMR spectroscopic data for compounds **1** and **2** in DMSO-*d*_6_.

No	**1**		**2**	
	*δ*_C_	*δ*_H_ (*J* in Hz)	*δ*_C_	*δ*_H_ (*J* in Hz)
1	64.1 C		64.2 C	
2	196.0 C		196.0 C	
3	112.5 C		112.5 C	
4	41.1 CH	3.15 t (2.8)	41.1 CH	3.12 t (2.6)
5	72.9 C		72.9 C	
6	209.6 C		209.6 C	
7	45.3 CH	3.11 dd (10.6, 5.8)	45.4 CH	3.05 dd (10.6, 5.8)
8 (a)	31.3 CH_2_	2.90 ddd (13.2, 10.6, 2.8)	31.4 CH_2_	2.88 ddd (13.4, 10.6, 2.8)
(b)		1.70 ddd (13.2, 5.8, 2.8)		1.68 ddd (13.4, 5.8, 2.8)
9	178.7 C		178.6 C	
10 (a)	31.5 CH_2_	2.67 dt (14.0, 7.5)	31.5 CH_2_	2.65 dt (14.0, 7.0)
(b)		2.48 dt (14.0, 6.7)		2.45 dt (14.0, 6.6)
11	28.9 CH_2_	2.32 m	28.9 CH_2_	2.33 m
12	129.6 CH	5.49 m (overlap)	129.6 CH	5.47 m (overlap)
13	125.9 CH	5.49 m (overlap)	125.9 CH	5.49 m (overlap)
14	17.7 CH_3_	1.56 d (4.7)	17.7 CH_3_	1.57 d (4.7)
15	134.3 C		132.8 C	
16	111.6 CH	6.53 d (1.5)	112.1 CH	6.49 d (1.8)
17	148.3 C		147.2 C	
18	147.8 C		145.5 C	
19	111.6 CH	6.83 d (8.2)	115.2 CH	6.63 d (8.1)
20	120.2 CH	6.51 dd (8.2, 1.5)	120.6 CH	6.37 dd (8.1, 1.8)
21	10.7 CH_3_	0.71 s	10.7 CH_3_	0.70 s
22	23.6 CH_3_	1.13 s	23.6 CH_3_	1.14 s
17-OMe	55.1 CH_3_	3.64 s	55.3 CH_3_	3.65 s
18-OMe	55.4 CH_3_	3.70 s		
5-OH		5.98 s		5.96 s
9-OH		14.68 br s		14.69 br s
18-OH				8.92 s

### ECD Calculation

As reported previously (Niu et al., 2020), conformational analyses were carried out *via* random searching in the Sybyl-X 2.0 using the MMFF94S force field with an energy cutoff of 2.0 kcal/mol. Subsequently, the lowest energy conformer was re-optimized using DFT at b3lyp/6-31+g(d,p) level in MeOH by the GAUSSIAN 09 program. The energies, oscillator strengths, and rotational strengths (velocity) of the first 30 electronic excitations were calculated using the TDDFT methodology at the cam-b3lyp/TZVP level using the polarizable continuum model in MeOH. The ECD spectrum were simulated by the overlapping Gaussian function (half the bandwidth at 1/e peak height, *σ* = 0.35, UV correction = 23 nm).

### Antiproliferative Bioassay

Briefly, four cancer cell lines of breast cancer cells (MCF-7), colon cancer cells (HT-29), hepatoma cells (HuH-7), and prostate cancer cells (LNCap) were treated with different concentrations of compounds **1**–**5** for 48 h. The cell viability was evaluated using MTT assay.

### Cell Cycle Experiment

HT-29 cells were treated with tested compounds. After 48 h, they were collected by trypsin and dehydrated with 70% EtOH overnight at 4°C. After alcohol was removed, cells were washed twice using PBS and were labeled by DAPI (1:10000 dilution in PBS, D8417 from Sigma-Aldrich, Saint Louis, MO, United States) for 10 min. Finally, fluorescence was measured by flow cytometry using PB450-A (CytoFLEX, Beckman Coulter, Kraemer Boulevard Brea, CA, United States).

### Western Blotting

Cell lysates were boiled in sodium dodecyl sulfate (SDS) sample loading buffer, resolved by 10% SDS-polyacrylamide gel electrophoresis (SDS-PAGE) and transferred to nitrocellulose. The membranes were blocked in 5% milk in Tris-buffered saline and Tween 20 [TBST; 10 mM Tris-HCl (pH 8.0), 150 mM NaCl, and 0.05% Tween 20] for 1 h at room temperature. After washing twice with TBST, the membranes were incubated with appropriate primary antibodies in TBST for 1 h and then washed twice, probed with horseradish peroxide-linked anti-immunoglobulin (1:5000 dilution) for 1 h at room temperature. After three washes with TBST, immunoreactive products were visualized using enhanced chemiluminescence reagents and autoradiography.

## Results and Discussion

**Figure 2:**
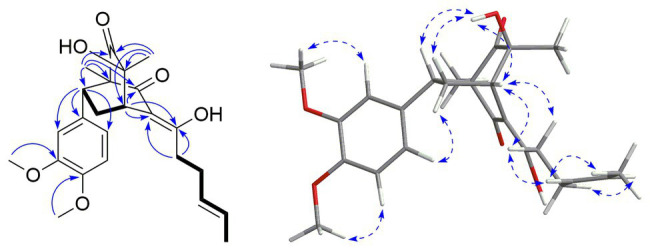
The Key COSY (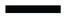), HMBC (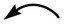), and NOESY (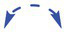) correlations of compound **1**.

Compound **1** was isolated as a yellow oil. The sodiated molecular ion peak at *m/z* 495.1951 [M + Na]^+^ in the HRESIMS indicated its molecular formula as C_24_H_30_O_6_, requiring 10 degrees of unsaturation. The ^1^H NMR spectrum showed two methyl singlets [*δ*_H_ 0.71 (3H, s, Me-21), 1.13 (3H, s, Me-22)], one methyl doublet [*δ*_H_ 1.56 (3H, d, *J* = 4.7 Hz, Me-14)], three typical ABX aromatic protons [*δ*_H_ 6.51 (1H, dd, *J* = 8.2, 1.5 Hz, H-20), 6.53 (1H, d, *J* = 1.5 Hz, H-16), 6.83 (1H, d, *J* = 8.2 Hz, H-19)], and two methoxyls [*δ*_H_ 3.64 (3H, s, 17-OMe), 3.70 (3H, s, 18-OMe)]. The ^13^C NMR spectrum indicated 24 carbon resonances including three methyls (*δ*_C_ 10.7, 17.7, and 23.6), three methylenes, seven methines (two olefinic and three aromatic), nine quaternary carbons (two ketones at *δ*_C_ 196.0 and 209.6, two olefinic at *δ*_C_ 112.5 and 178.7, and three aromatic at *δ*_C_ 134.3, 147.8, and 148.3), and two methoxyls. Since the ABX aromatic moiety, two ketones, and the other four olefinic carbons accounted for eight unsaturations, compound **1** was deduced to be a bicyclic molecule. In the ^1^H–^1^H COSY spectrum, two segments could easily be deduced on the basis of correlations of H_2_-8 to H-4/H-7 and Me-14 *via* H-12/H-13 to H_2_-11/Hab-10 (Figure 2). These two segments along with the ABX aromatic moiety could be connected by the HMBC correlations of Me-21 to C-1 (*δ*_C_ 64.1 s)/C-2 (*δ*_C_ 196.0 s)/C-6 (*δ*_C_ 209.6 s)/C-7 (*δ*_C_ 45.3 d), Me-22 to C-4 (*δ*_C_ 41.1 d)/C-5 (*δ*_C_ 72.9 s)/C-6, H-4 (*δ*_H_ 3.15, t, *J* = 2.8 Hz) to C-2/C-3 (*δ*_C_ 112.5 s)/C-9 (*δ*_C_ 178.7 s), H-7 (*δ*_H_ 3.11, dd, *J* = 10.6, 5.8 Hz) to C-15 (*δ*_C_ 134.3 s)/C-16 (*δ*_C_ 111.6 d)/C-20 (*δ*_C_ 120.2 d), Hab-10 (*δ*_H_ 2.67, dt, *J* = 14.0, 7.5 Hz; 2.48, dt, *J* = 14.0, 6.7 Hz) to C-3/C-9, 17-OMe to C-17 (*δ*_C_ 148.3 s), and 18-OMe to C-18 (*δ*_C_ 147.8 s). Accordingly, the planar structure of compound **1** was established as a sorbicillinoid derivate, structurally related to sorbicatechol A. In the NOESY spectrum, 5-OH (*δ*_H_ 5.98 s) was correlated to H-7 (*δ*_H_ 3.11, dd, *J* = 10.6, 5.8 Hz)/Ha-8 (*δ*_H_ 2.90, ddd, *J* = 13.2, 10.6, 2.8 Hz), while Hb-8 (*δ*_H_ 1.70, ddd, *J* = 13.2, 5.8, 2.8 Hz) was correlated to H-16/H-20. This suggested co-plane of H-7 and 5-OH. Furthermore, correlations of Hab-10 (*δ*_H_ 2.67, dt, *J* = 14.0, 7.5 Hz, Ha-10; 2.48, dt, *J* = 14.0, 6.7 Hz, Hb-10) to H-4/H-12(H-13) deduced the *Z*-orientation of the olefinic bonds of C-3/C-9. Since the chemical shifts of H-12 and H-13 were overlapped, it was impossible to assign the configuration by their coupling constants. However, the NOESY correlations of Me-14 to H-12(H-13) but not H_2_-11 could suggest the *E*-orientation of C-12/C-13. On the basis of the above evidences, the relative configuration of compound **1** was, therefore, determined undoubtedly.

**Figure 3:**
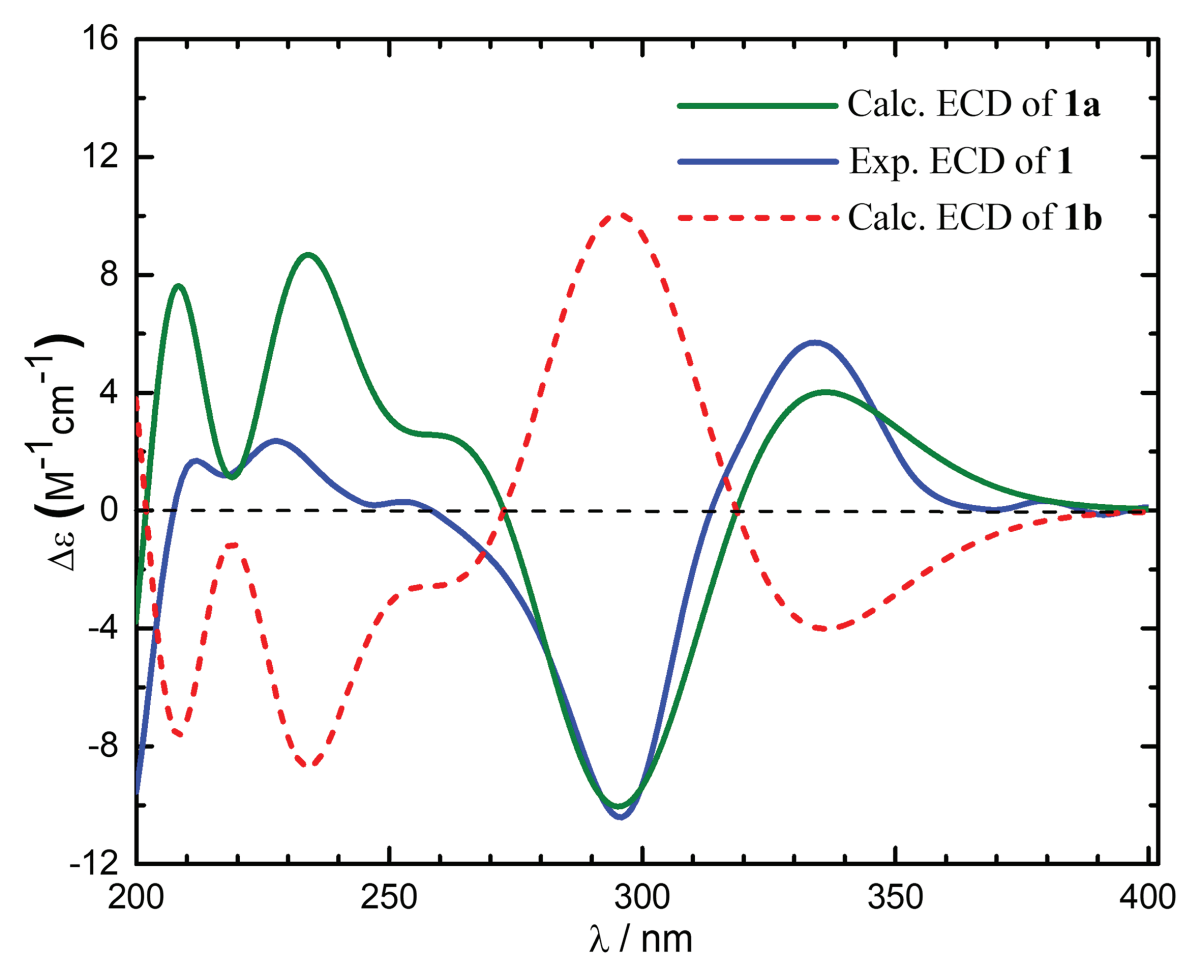
Experimental and calculated electronic circular dichroism (ECD) spectra of compound **1** in MeOH.

To further assign its absolute stereochemistry, the theoretical calculation of the electronic circular dichroism (ECD) was conducted. As shown in Figure 3, the experimental ECD spectrum of compound **1** showed the same Cotton effects as those of 1*R,*4*S,*5*S,*7*R*-**1** (**1a**). Therefore, the absolute stereochemistry of compound **1** was assigned as 10,11-dihydrosorbicatechol A, and named sorbicatechol C.

Compound **2** was also isolated as a yellow oil. The molecular formula was assigned as C_23_H_28_O_6_ on the basis of the HRESIMS at *m/z* 399.1790 [M−H]^−^ (calcd for C_23_H_27_O_6_, 399.1808). The ^1^H and ^13^C NMR spectroscopic data (Table 1) indicated 23 carbon resonances including two methyl singlets, one methyl doublet, three methylenes, seven methines (two olefinic and three aromatic), nine quaternary carbons (two ketones at *δ*_C_ 196.0 and 209.6, two olefinics at *δ*_C_ 112.5 and 178.6, and three aromatics at *δ*_C_ 132.8, 145.5, and 147.2), and one methoxyl. These signals were very similar to those of compound **1**, except that the methoxyl group at the C-18 position in compound **1** was changed to a hydroxy moiety in compound **2**. This was evidenced by an additional hydroxy unit and the absence of a methoxyl group in compound **2**. Further confirmation could be found by the HMBC correlations of the hydroxy at *δ*_H_ 8.92 s to C-19 (*δ*_C_ 115.2 d). By detailed analysis of its heteronuclear single quantum correlations (HSQC), correlation spectroscopy (COSY), heteronuclear multiple-bond correlation (HMBC), and nuclear overhauser effect (NOESY) spectroscopic data (Supplementary Table S1 of the Supporting Information), the structure and relative configuration of the compound **2** were determined. The absolute configuration of compound **2** was assigned to be identical to that of compound **1** based on their similar specific rotations and ECD data. Compound **2** was then established as 18-*O*-demethyl derivate of sorbiallisatol A, and named sorbicatechol D.

Hybrid sorbicillinoids are derived from either a Diels-Alder or a Michael reaction of a monomeric sorbicillinoid diene and a second non-sorbicillinoid dienophile (Meng et al., 2016). Noteworthily, compounds **1** and **2** are two novel hybrid sorbicillinoids derived from dihydrosorbicillinols which were very rarely in nature. Their biogenetic origin might be involved ferulic acid and 2',3'-dihydrosorbicillinol *via* an intermolecular Diels-Alder condensation followed by a decarboxylation (Peng et al., 2014).

By comparison of NMR spectroscopic data with those published in the literatures, three known compounds were determined to be sohirnone A (**3**), 2',3'-dihydrosorbicillin (**4**; Maskey et al., 2005), and sorbicillin (**5**; Trifonov et al., 1983).

**Figure 4:**
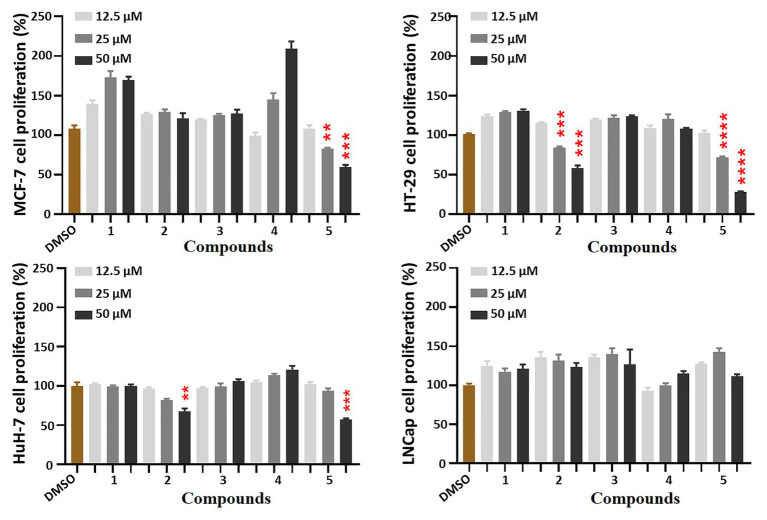
Antiproliferative effects of compounds **1**–**5** against four cancer cells. Cells were treated with five compounds in a dose-course (12.5, 25, and 50 μM) for 48 h, which were analyzed by MTT assays to compare the cell proliferation levels. All the bar graphs represent mean ± SEM of three independent experiments. ^**^p < 0.01, ^***^p < 0.001, ^****^p < 0.0001 vs. the DMSO group.

All five isolates were subjected to the preliminary screening tests for antiproliferative activity against MCF-7, HT-29, HuH-7, and LNCap tumor cells. Compounds **2** and **5** could inhibit the proliferation of HT-29 cells in a dose-dependent manner (Figure 4).

**Figure 5:**
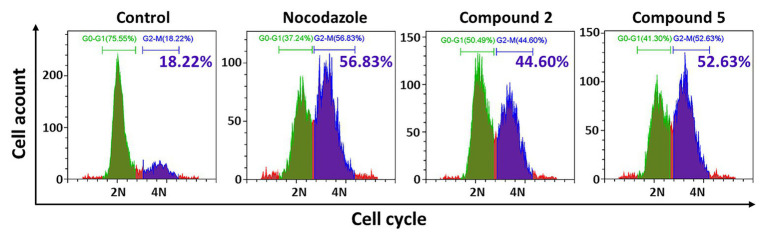
Effects of compounds **2** and **5** on cell cycle by flow cytometry. HT-29 cells were treated with compound **2** (50 μM, 48 h) or **5** (30 μM, 48 h), which were stained with DAPI for the following cell cycle FCM assay. The blue part indicated the cells in G0-G1 phase, the green part showed the G2-M phase cells, and the green numbers showed the G2-M phase proportion.

Interestingly, after treatment by compounds **2** and **5**, many HT-29 cells became more rounded and less adherent under the microscope, which suggested cell cycle was disrupted and resulted in M-phase arresting. Therefore, further investigation of the cell cycle was conducted by flow cytometry. As expected, they significantly blocked 40.96 and 41.69% tumor cells in G2-M phase, respectively (Figure 5). Therefore, compounds **2** and **5** could blocked HT-29 cells in G2-M phase.

**Figure 6:**
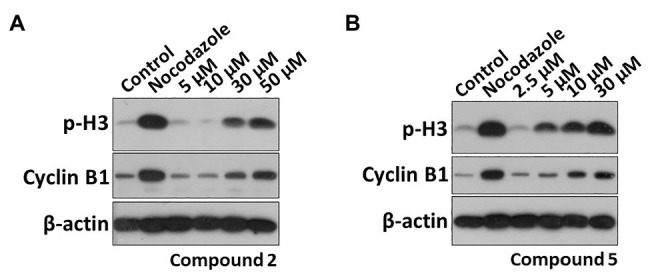
Effects of compounds **2** and **5** on the expression of M phase marker proteins. HT29 cells were treated with **(A)** compound **2** (5, 10, 30, and 50 μM) or **(B)** compound **5** (2.5, 5, 10, and 30 μM) for 48 h. The p-H3 and Cyclin B1 protein levels were analyzed by Western blot (WB).

Phosphorylation of histone H3 (p-H3) is one of the methods of histone modification. It occurs at specific periods and chromosomal sites during mitosis and meiosis. Cyclin B1 also plays an important role in cell cycle regulation. The overexpression of cyclinB1 can promote G2/M phase conversion and even lead to uncontrolled cell proliferation and malignant transformation (Hartwell and Kastan, 1994; Hwang et al., 1995). Therefore, the expression of M phase markers p-H3 and cyclin B1 was further detected. As shown in Figure 6, they could significantly increase the protein levels of p-H3 and cyclin B1 in a dose-dependent effect, confirming compounds **2** and **5** indeed induced M phase arresting. The effective concentrations of compounds **2** and **5** were 30 and 5 μM, respectively.

## Conclusion

From the deep-sea-derived *Penicillium allii-sativi* MCCC 3A00580, five sorbicillinoids (**1**–**5**) were obtained. Compounds **1** and **2** are two novel hybrid dihyrosorbicillinoids. While compounds **3**–**5** are three known monomeric sorbicillinoids. Although several examples were found for monomeric, dimeric, and trimeric sorbicillinoids in which C-2'/C-3' double bonds were reduced, compounds **1** and **2** are the first two examples for hybrid sorbicillinoids. Therefore, the discovery of compounds **1** and **2** has expanded the diversity and complexity of sorbicillinoids. Compounds **2** and **5** could inhibit HT-29 tumor cells in a good dose-dependent manner. They significantly induced cell cycle G2-M phase arresting by increasing the protein levels of p-H3 and cyclin B1.

## Data Availability Statement

The raw data supporting the conclusions of this article will be made available by the authors, without undue reservation.

## Author Contributions

C-LX performed chemical investigations. DZ conducted biological experiments. TL, Z-HH, and Q-XY assisted C-LX’s chemical experiments. QC assisted DZ’s bioactive experiments. X-KZ, X-WY, and H-FC initiated and oversaw all research. All authors contributed to the article and approved the submitted version.

### Conflict of Interest

The authors declare that the research was conducted in the absence of any commercial or financial relationships that could be construed as a potential conflict of interest.
