# Supplementary material for: Antiproliferative Sorbicillinoids From the Deep-Sea-Derived Penicillium allii-sativi
Source: Front Microbiol. 2021 Jan 21;11:636948. doi: 10.3389/fmicb.2020.636948 (PMC7858254; doi:10.3389/fmicb.2020.636948)
Supplement: Supplementary file 1 [file Data_Sheet_1.pdf]

Supplementary Material

Supplementary Table S1. Optimized cartesian coordinates for the lowest energy conformer reoptimized at b3lyp/6-31+g(d,p) level in MeOH for 1*R*, 4*S*, 5*S*, 7*R*-1 (**1a**).

Supplementary Figure S1. ^{1}H-NMR (400 MHz, DMSO-d_6) spectrum of compound **1**

Supplementary Figure S2. ^{13}C-NMR (100 MHz, DMSO-d_6) spectrum of compound **1**

Supplementary Figure S3. HSQC spectrum of compound **1**

Supplementary Figure S4. ^{1}H–^{1}H COSY spectrum of compound **1**

Supplementary Figure S5. HMBC spectrum of compound **1**

Supplementary Figure S6. NOESY spectrum of compound **1**

Supplementary Figure S7. ^{1}H-NMR (400 MHz, DMSO-d_6) spectrum of compound **2**

Supplementary Figure S8. ^{13}C-NMR (100 MHz, DMSO-d_6) spectrum of compound **2**

Supplementary Figure S9. HSQC NMR spectrum of compound **2**

Supplementary Figure S10. ^{1}H–^{1}H COSY NMR spectrum of compound **2**

Supplementary Figure S11. HMBC NMR spectrum of compound **2**

Supplementary Figure S12. NOESY NMR spectrum of compound **2**.

Supplementary Table S1. Optimized cartesian coordinates for the lowest energy conformer reoptimized at b3lyp/6-31+g(d,p) level in MeOH for 1*R*, 4*S*, 5*S*, 7*R*-**1** (**1a**).

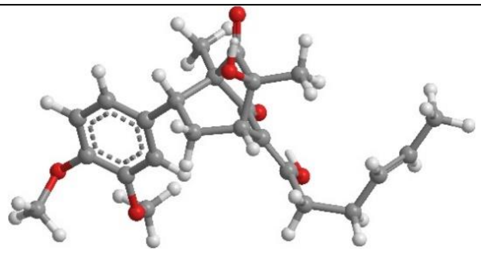

C	-1.90626500	-1.52676900	-0.27399300		C	4.05793200	-2.96347700	1.07239600
O	-1.74043600	-2.37277300	0.74394300		H	-1.22051500	-1.88090000	1.45318400
C	-2.62733900	-2.13627900	-1.44212400		H	-2.81987400	-1.37831500	-2.20323700
C	-3.94529700	-2.85947100	-1.06991000		H	-1.94281500	-2.87011200	-1.88947700
C	-5.02661100	-1.93301700	-0.57949100		H	-4.28629500	-3.37244900	-1.97890100
C	-5.65207800	-2.03678500	0.60023000		H	-3.74357200	-3.63351600	-0.32297100
C	-6.74626300	-1.12781400	1.08637100		H	-5.31233700	-1.12698100	-1.25902900
C	-1.39124000	-0.24656100	-0.20342100		H	-5.36090200	-2.84302300	1.27632800
C	-1.43196600	0.82580800	-1.27638900		H	-6.46670600	-0.64311000	2.03048200
C	0.02988100	1.13823800	-1.68031900		H	-6.97483300	-0.34612700	0.35492200
C	-0.64148100	0.15505300	0.96264600		H	-7.66738600	-1.69041600	1.28530800
C	0.83867300	1.65837700	-0.45341200		H	-2.00494700	0.51439900	-2.15164000
C	-0.06116900	1.56917000	0.87721400		H	0.47831500	0.22582800	-2.08034400
C	-1.23842000	2.50111700	0.55230700		H	0.04963500	1.88671800	-2.47535900
O	-1.46350100	3.53243600	1.16226200		H	-3.68623700	1.11219300	0.44008600
C	-2.08316300	2.09633800	-0.67975100		H	-3.94693700	2.83815300	0.13403700
C	-3.55356200	1.91081900	-0.29364400		H	-4.13005000	1.66153100	-1.18947800
O	-1.95932000	3.14379500	-1.65375100		H	-2.30164400	3.96117000	-1.26091200
O	-0.45775600	-0.57091500	1.97016300		H	1.48564800	1.30149200	2.36606800
C	0.67767400	1.99827100	2.13764700		H	1.10207600	2.99707600	2.00786500
H	1.01670200	2.72995800	-0.58597900		H	-0.00282500	2.02683800	2.99219000
C	2.20582900	1.01465700	-0.28648300		H	1.54370800	-1.05352300	-0.42308800
C	2.38332600	-0.37801200	-0.29251100		H	5.48759700	1.88665200	0.14207400
C	3.64514900	-0.95256000	-0.13368000		H	3.23524000	2.90270700	-0.10808500
C	4.77830400	-0.12748000	0.01192000		H	6.00770400	-2.08263900	-1.35286400
C	4.61006000	1.25765700	0.02696500		H	6.74033300	-0.50166400	-1.77652000
C	3.33988000	1.82148600	-0.11718100		H	7.60233100	-1.62479700	-0.68589100
O	6.03897600	-0.65749900	0.18670300		H	4.13060800	-4.03130200	0.86130800
C	6.62197500	-1.25421100	-0.98865400		H	5.00520200	-2.60039500	1.48127900
O	3.76100800	-2.32611500	-0.18560100		H	3.24801000	-2.78420100	1.78849000

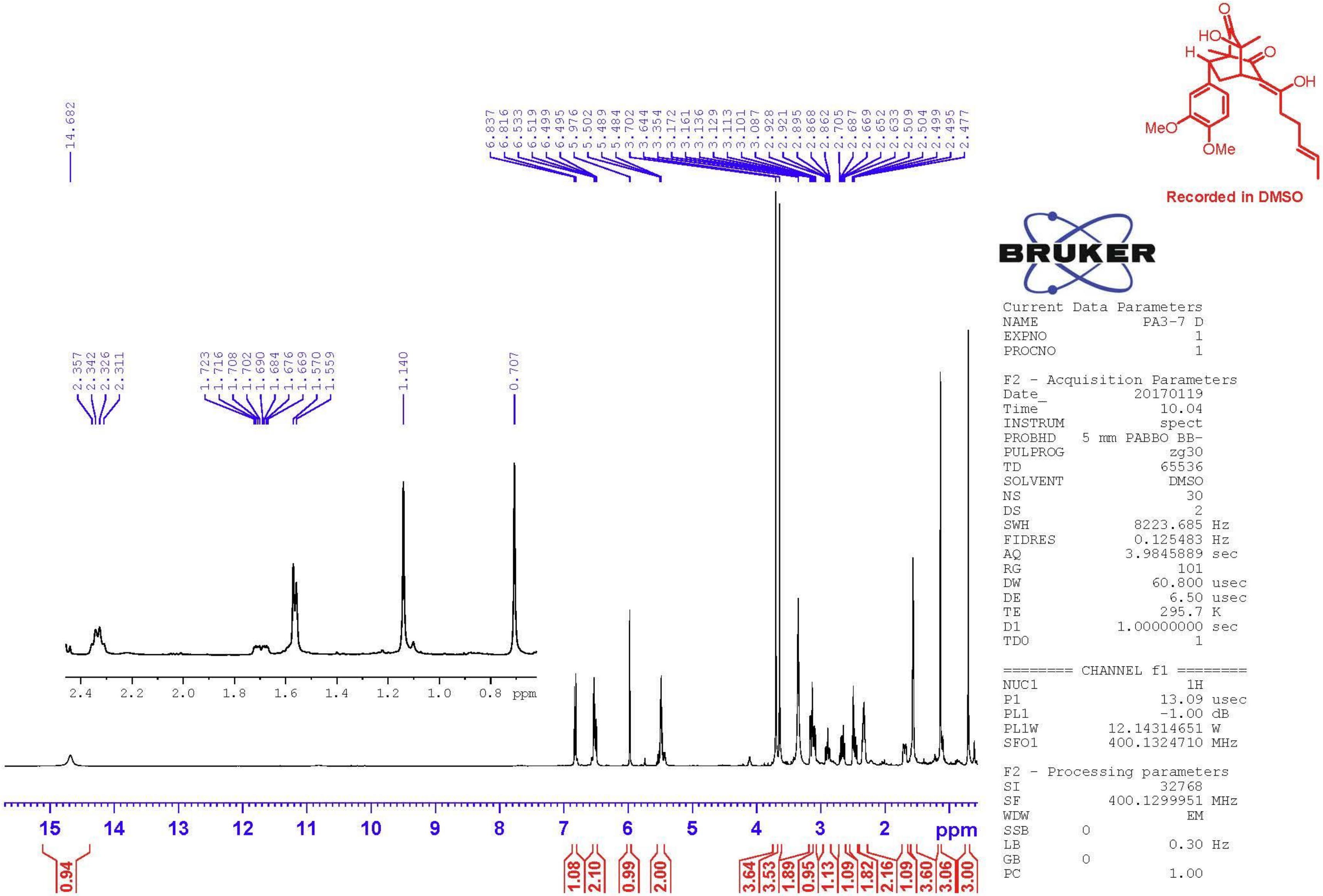

Supplementary Figure S1. ^{1}H-NMR (400 MHz, DMSO-d_6) spectrum of compound **1**

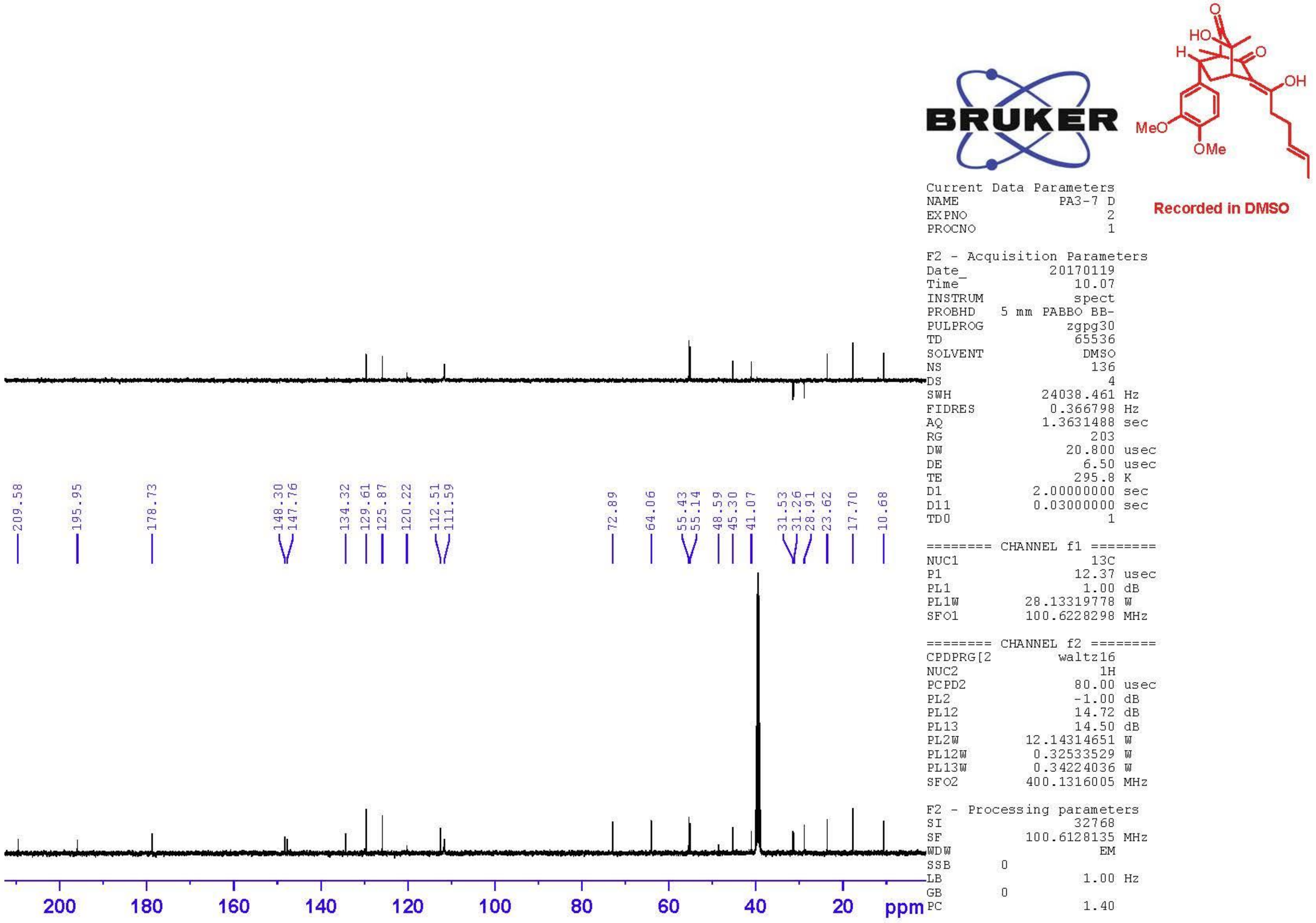

Supplementary Figure S2. ^{13}C-NMR (100 MHz, DMSO-d_6) spectrum of compound **1**

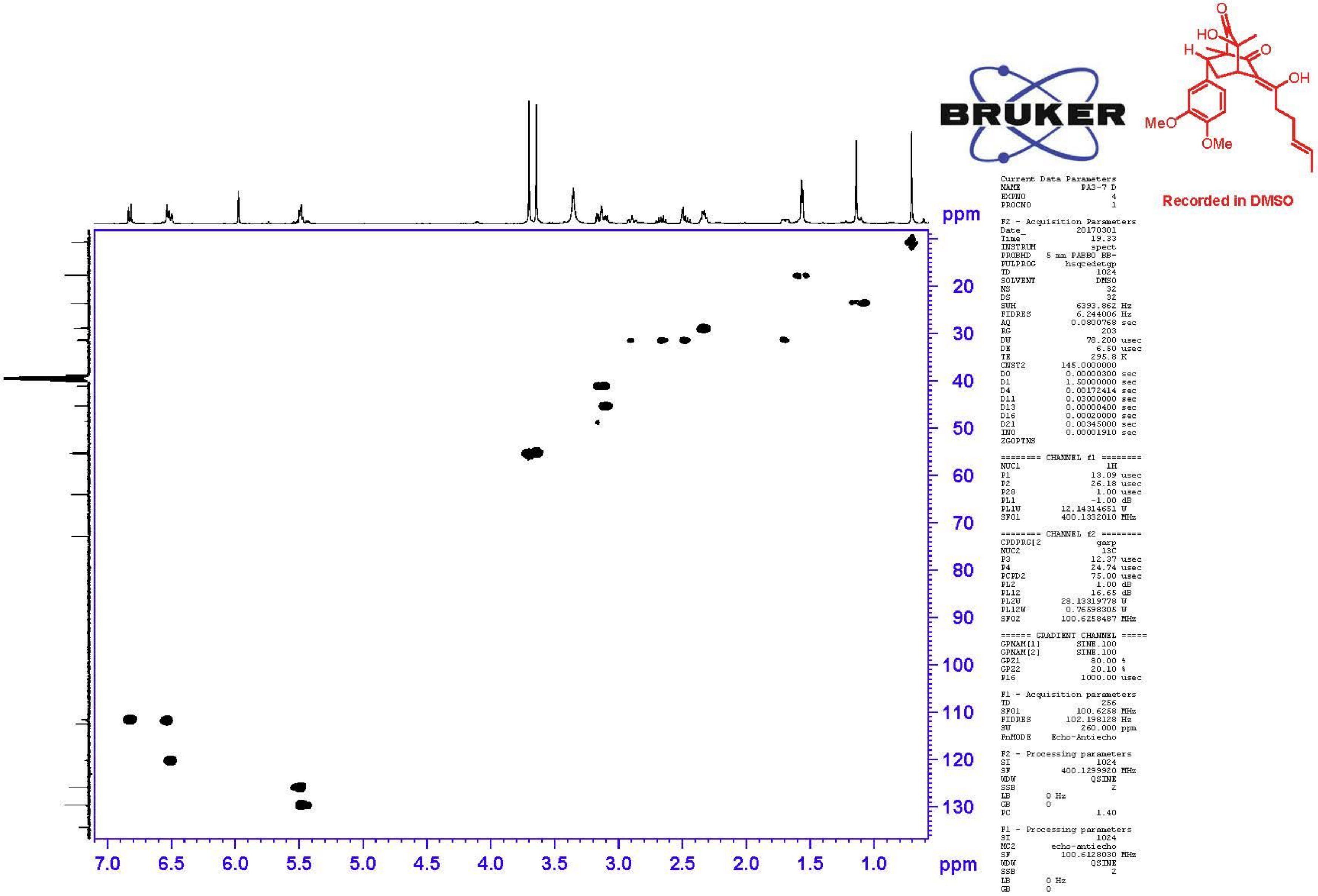

Supplementary Figure S3. HSQC spectrum of compound **1**

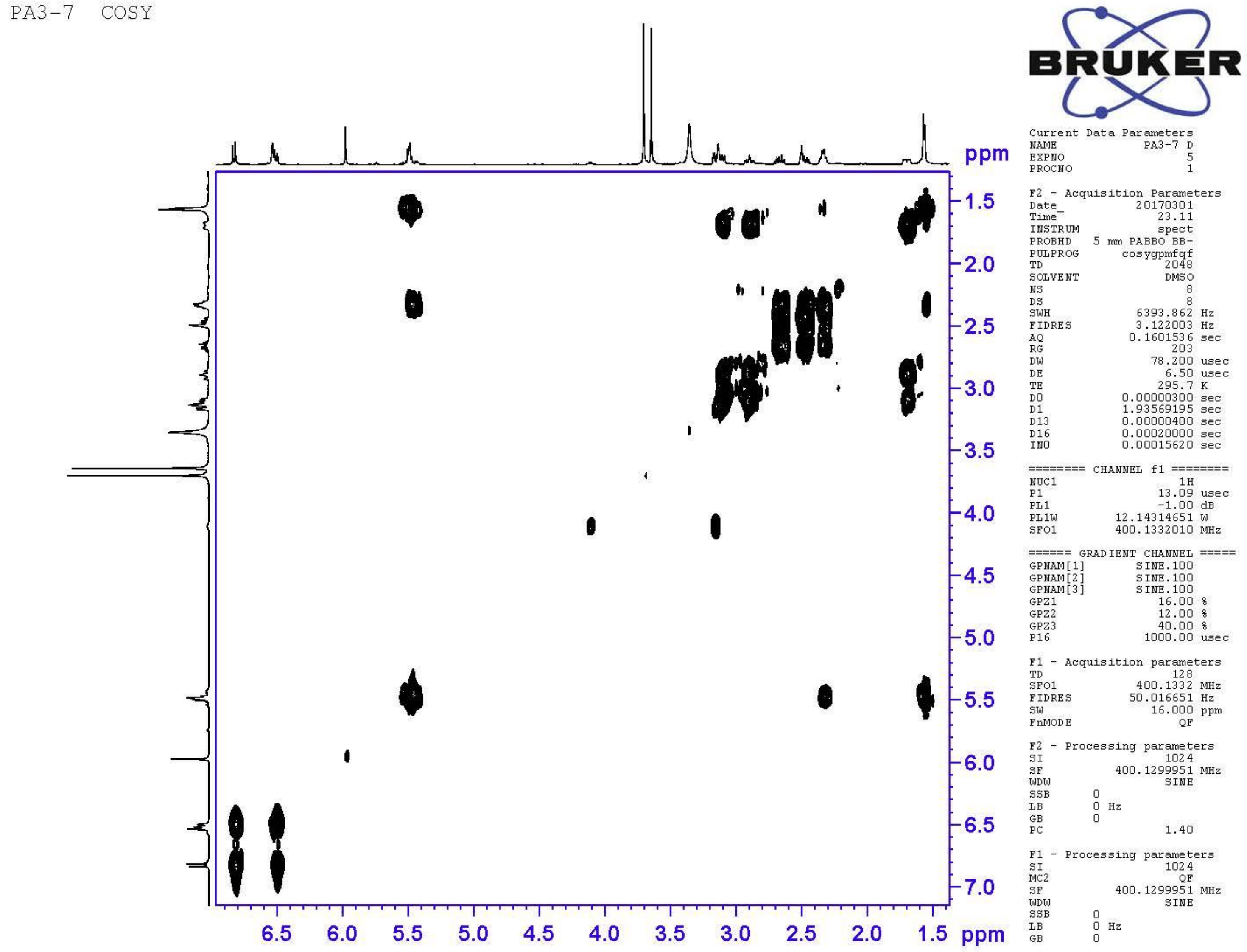

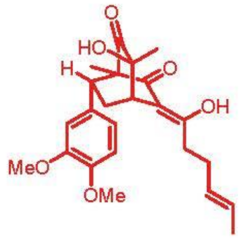

Recorded in DMSO

Supplementary Figure S4. ^1H-^1H COSY spectrum of compound **1**

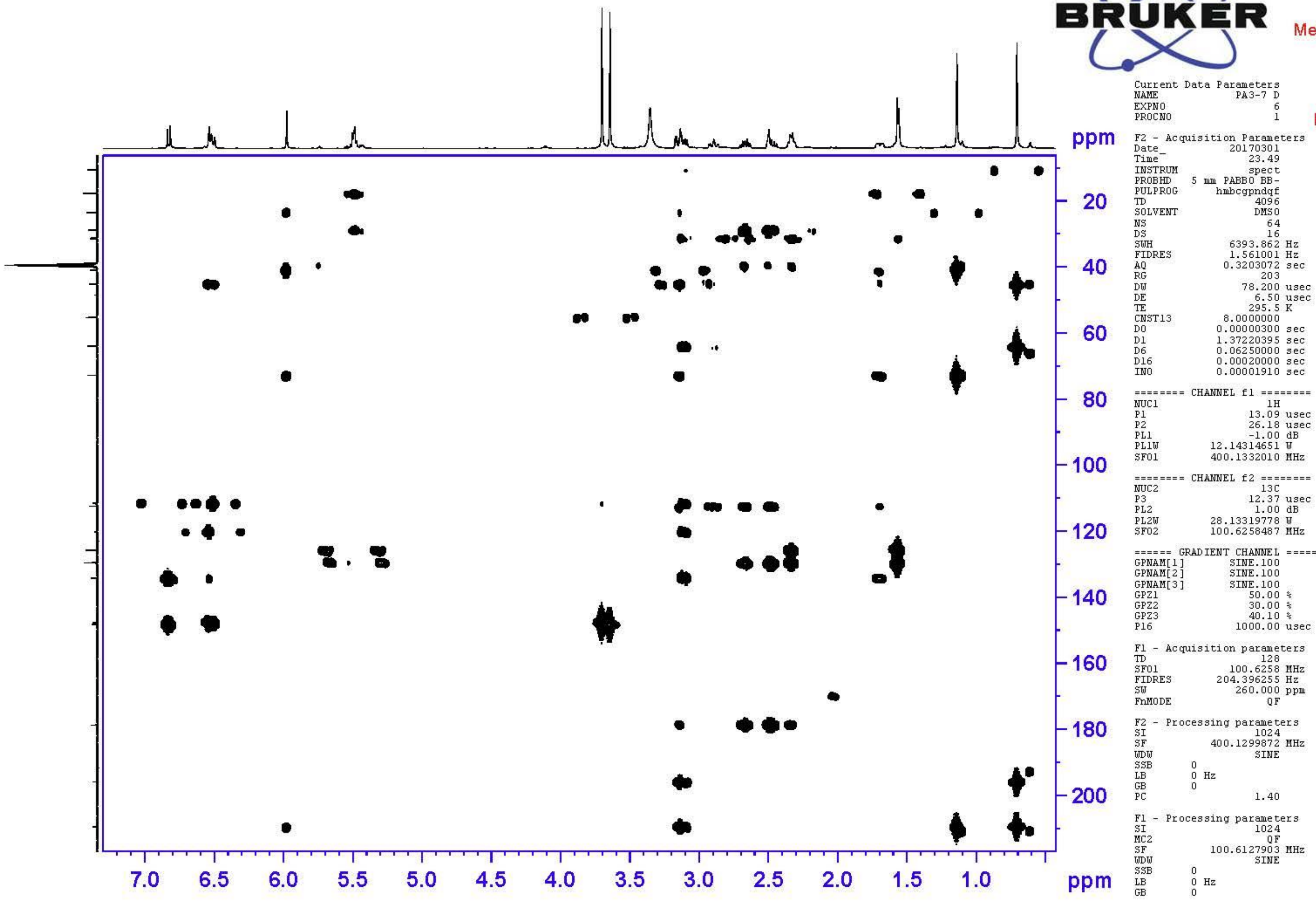

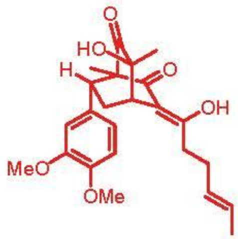

Supplementary Figure S5. HMBC spectrum of compound **1**

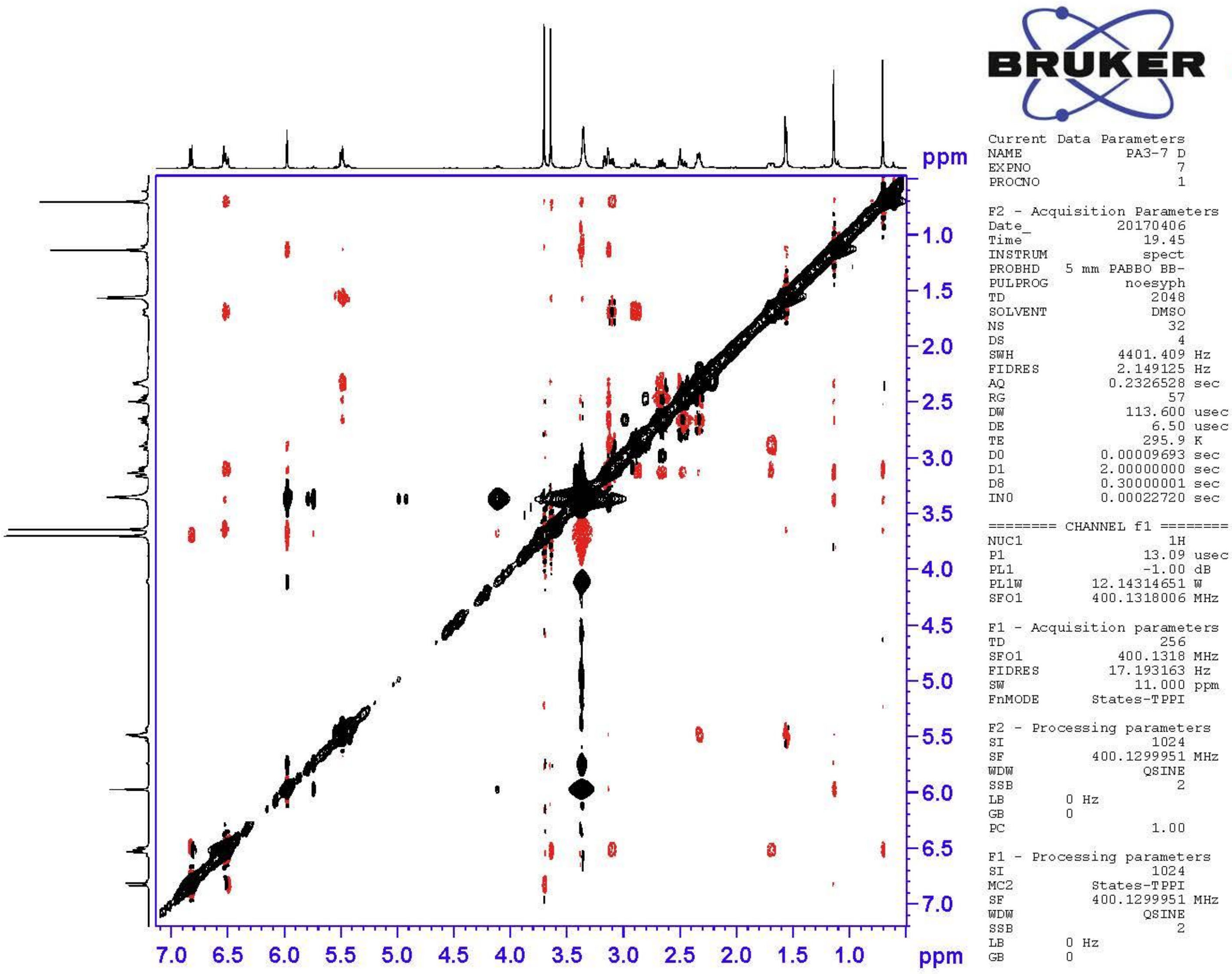

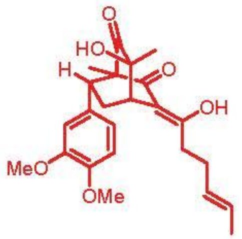

Recorded in DMSO

Supplementary Figure S6. NOESY spectrum of compound **1**

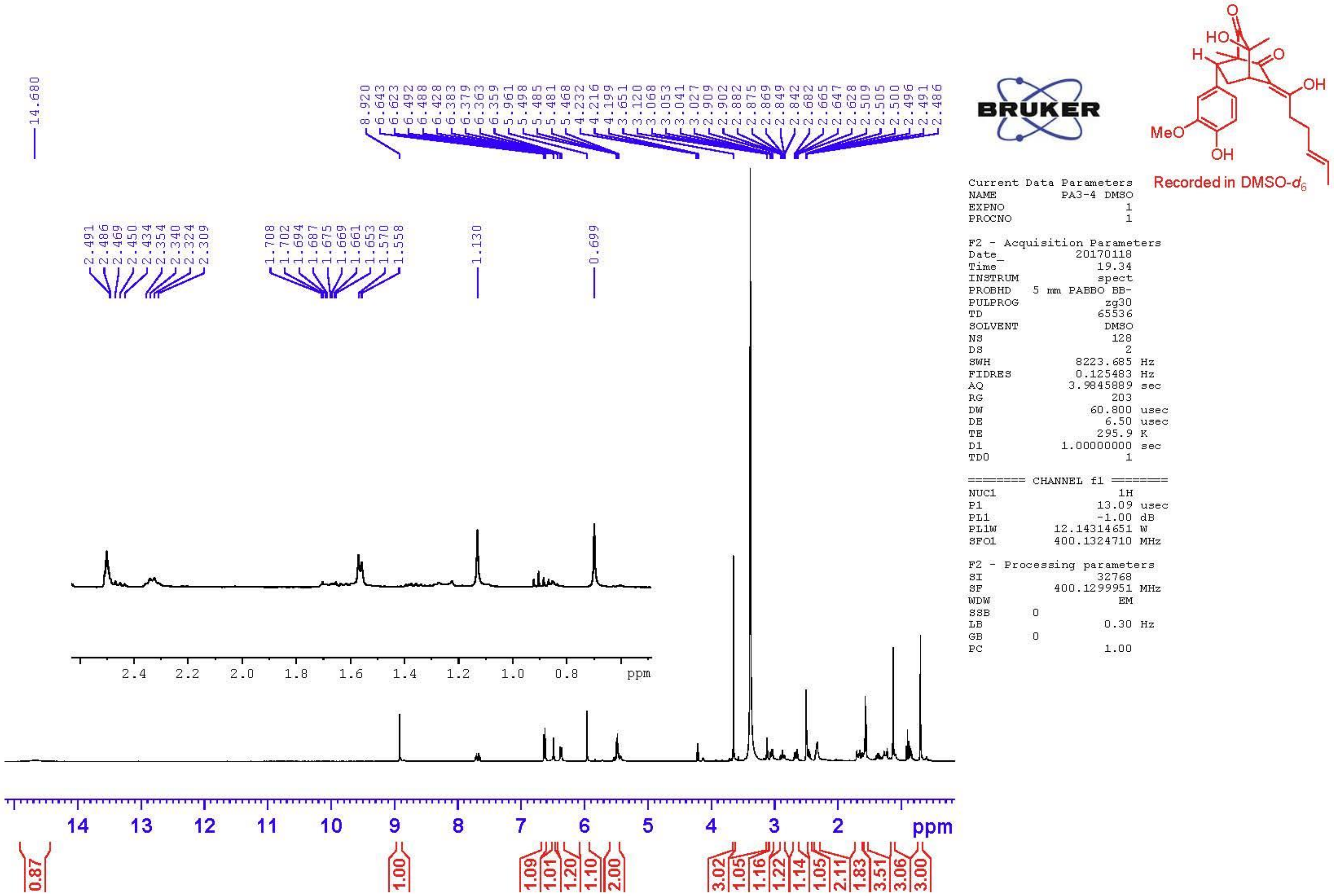

Supplementary Figure S7. ^{1}H-NMR (400 MHz, DMSO-d_6) spectrum of compound **2**

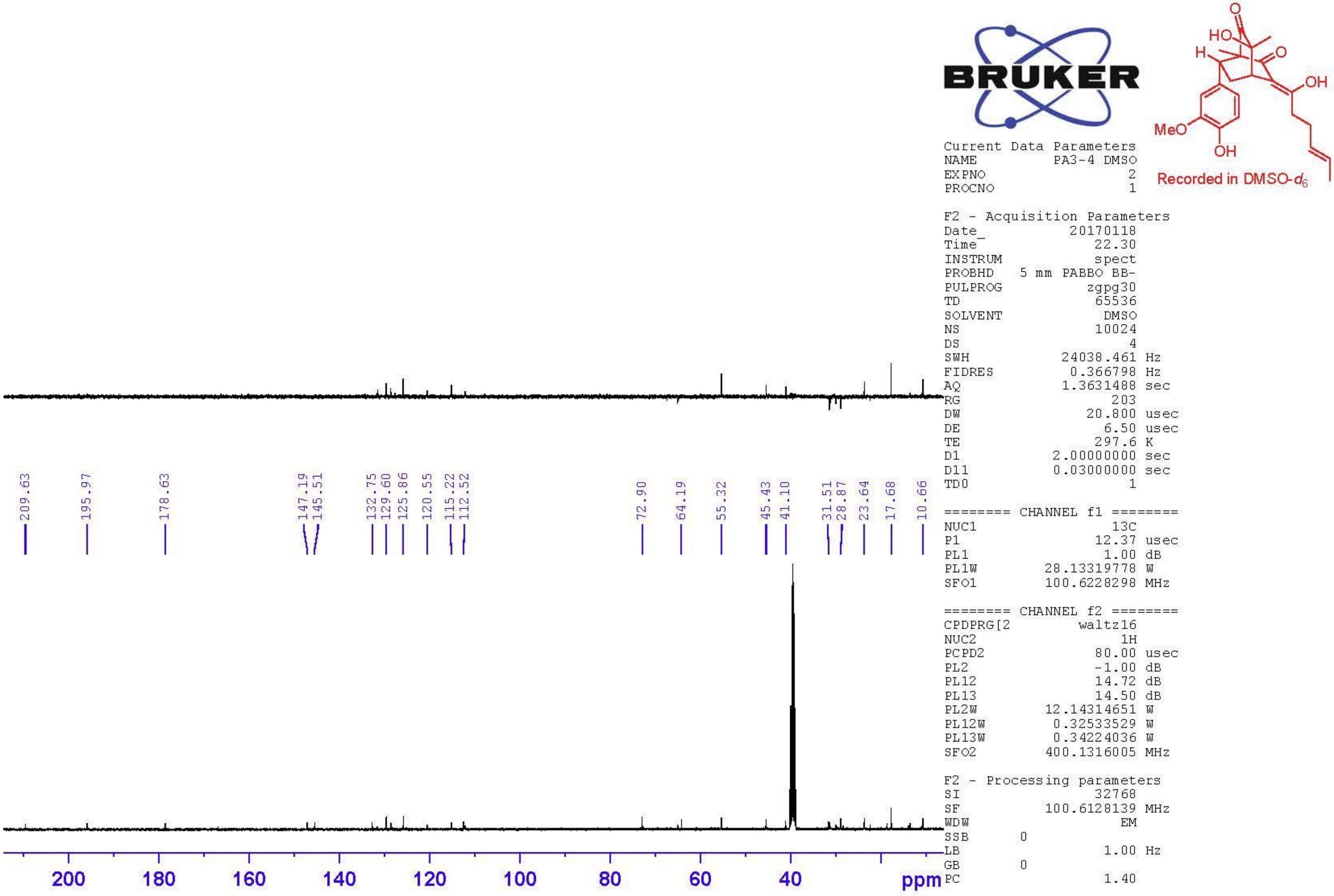

Supplementary Figure S8. ^{13}C-NMR (100 MHz, DMSO-d_6) spectrum of compound **2**

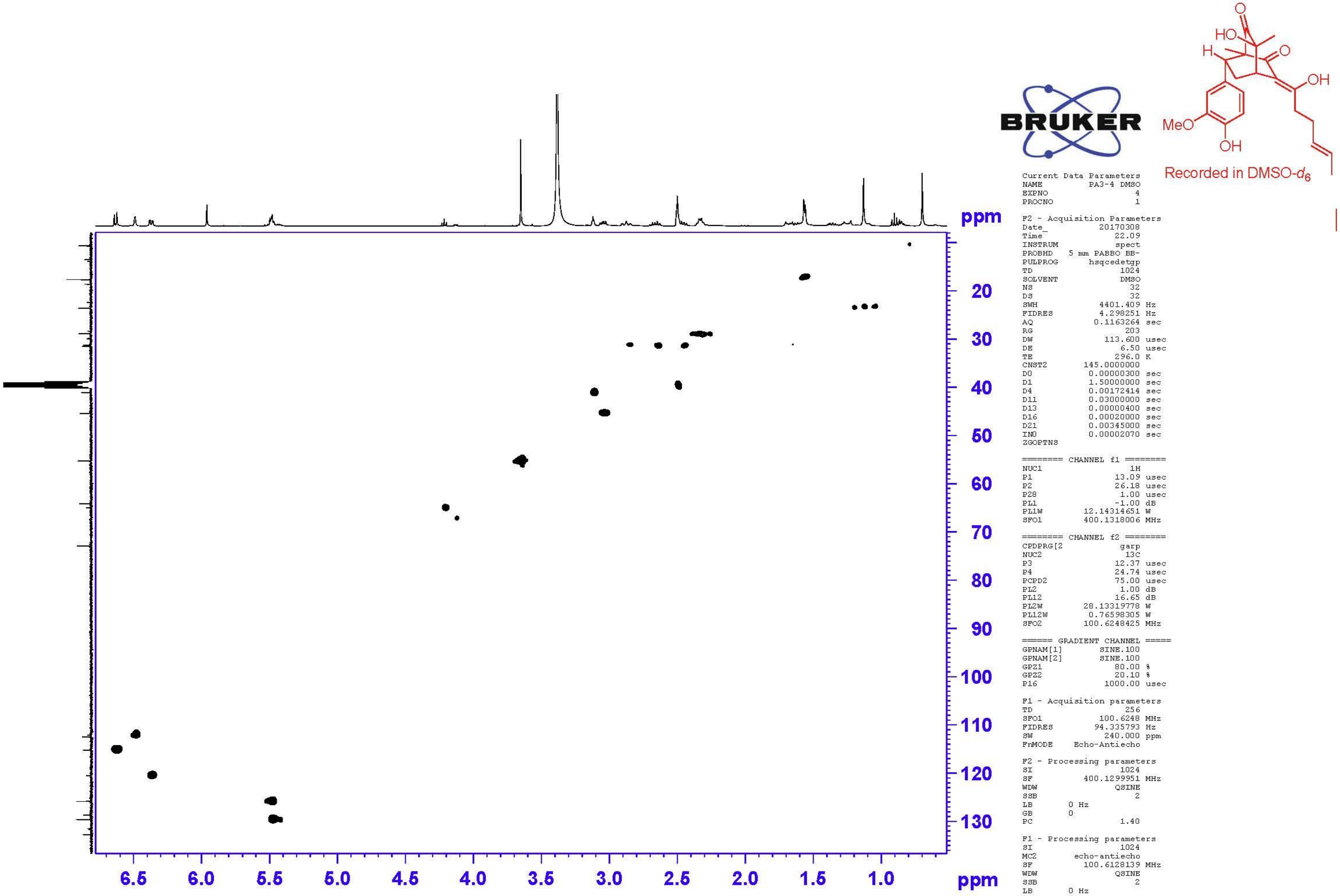

Supplementary Figure S9. HSQC NMR spectrum of compound **2**

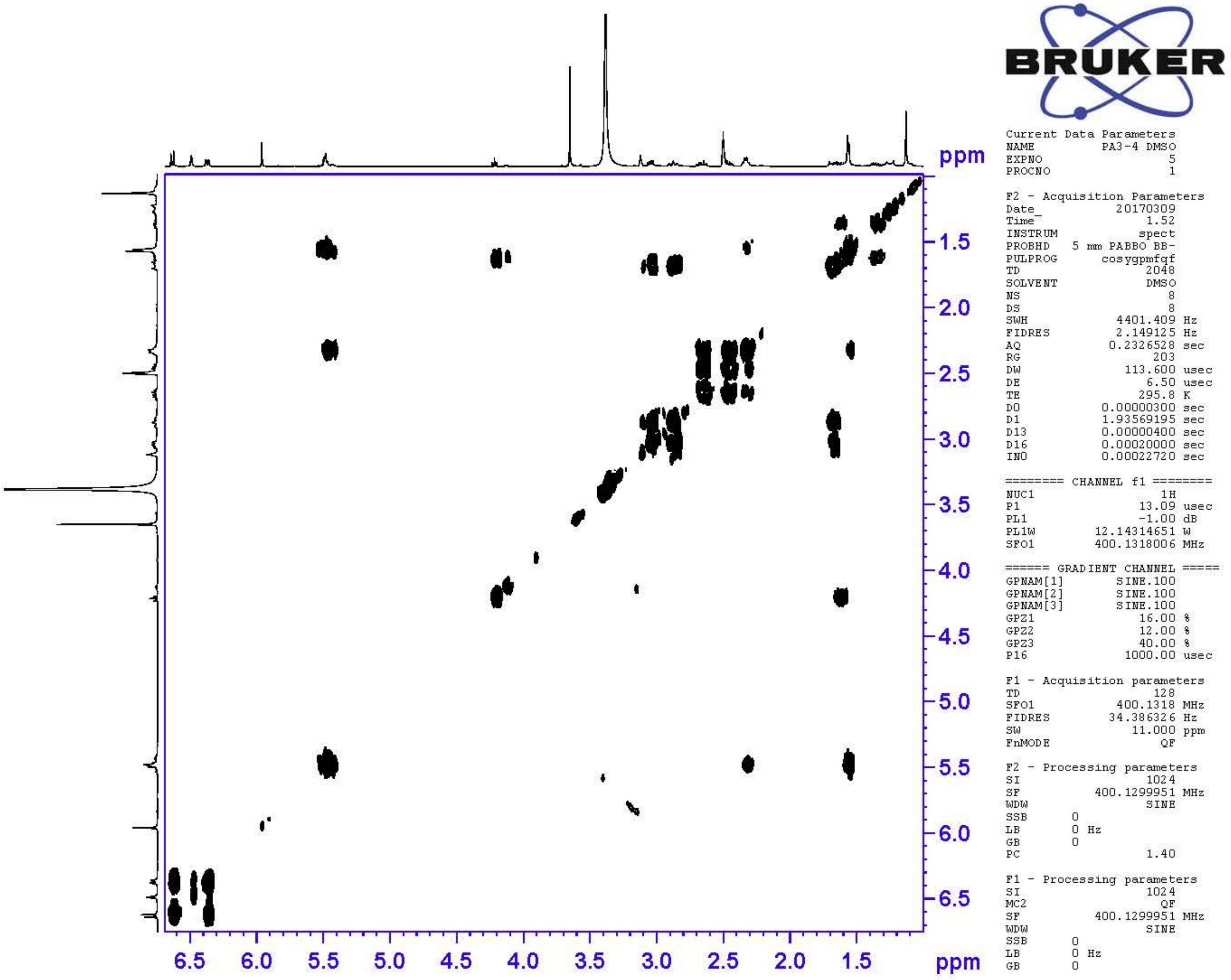

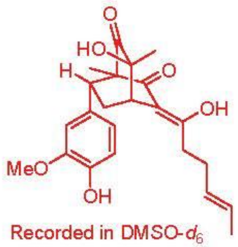

Supplementary Figure S10. ^{1}H -^{1}H COSY NMR spectrum of compound **2**

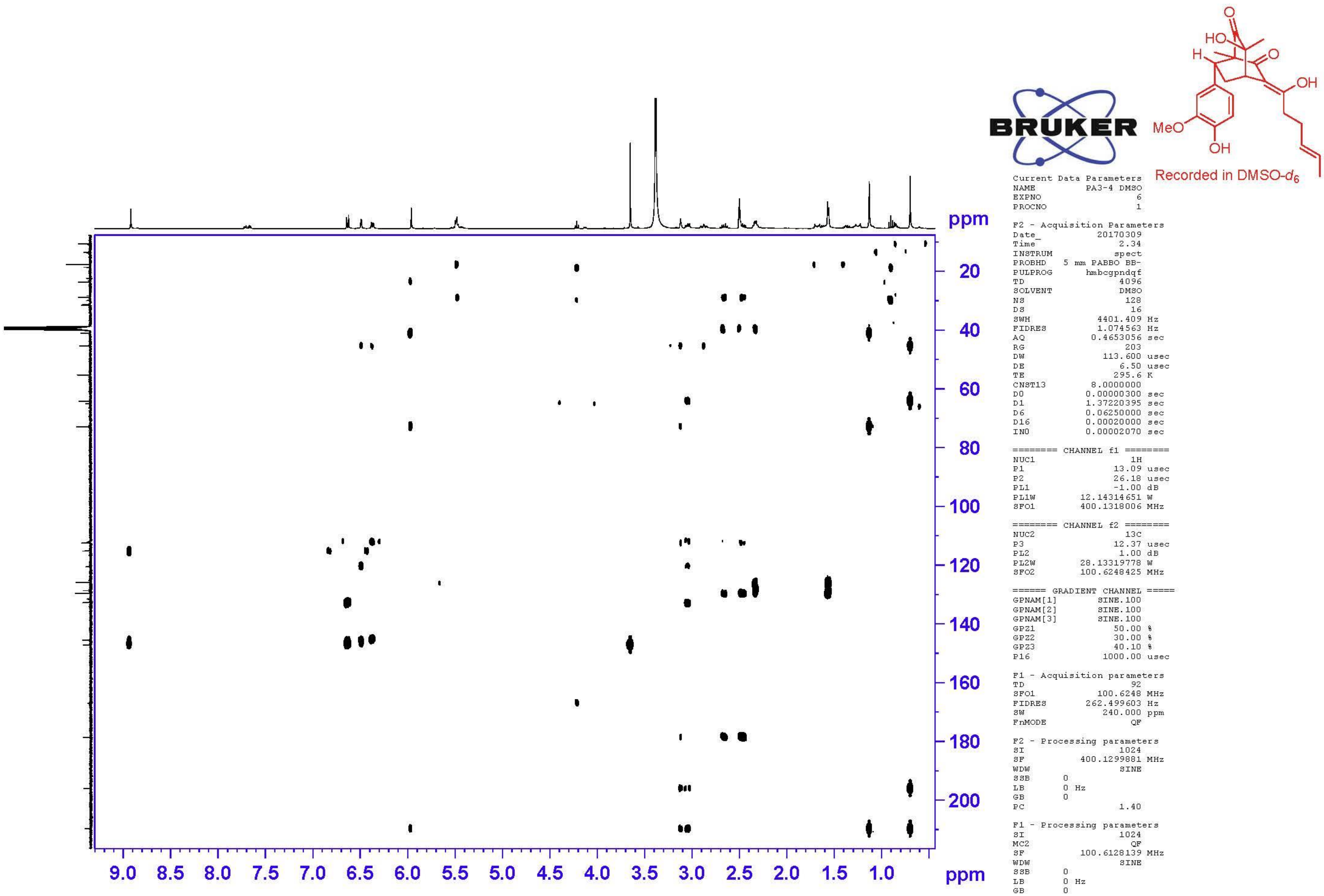

Supplementary Figure S11. HMBC NMR spectrum of compound **2**

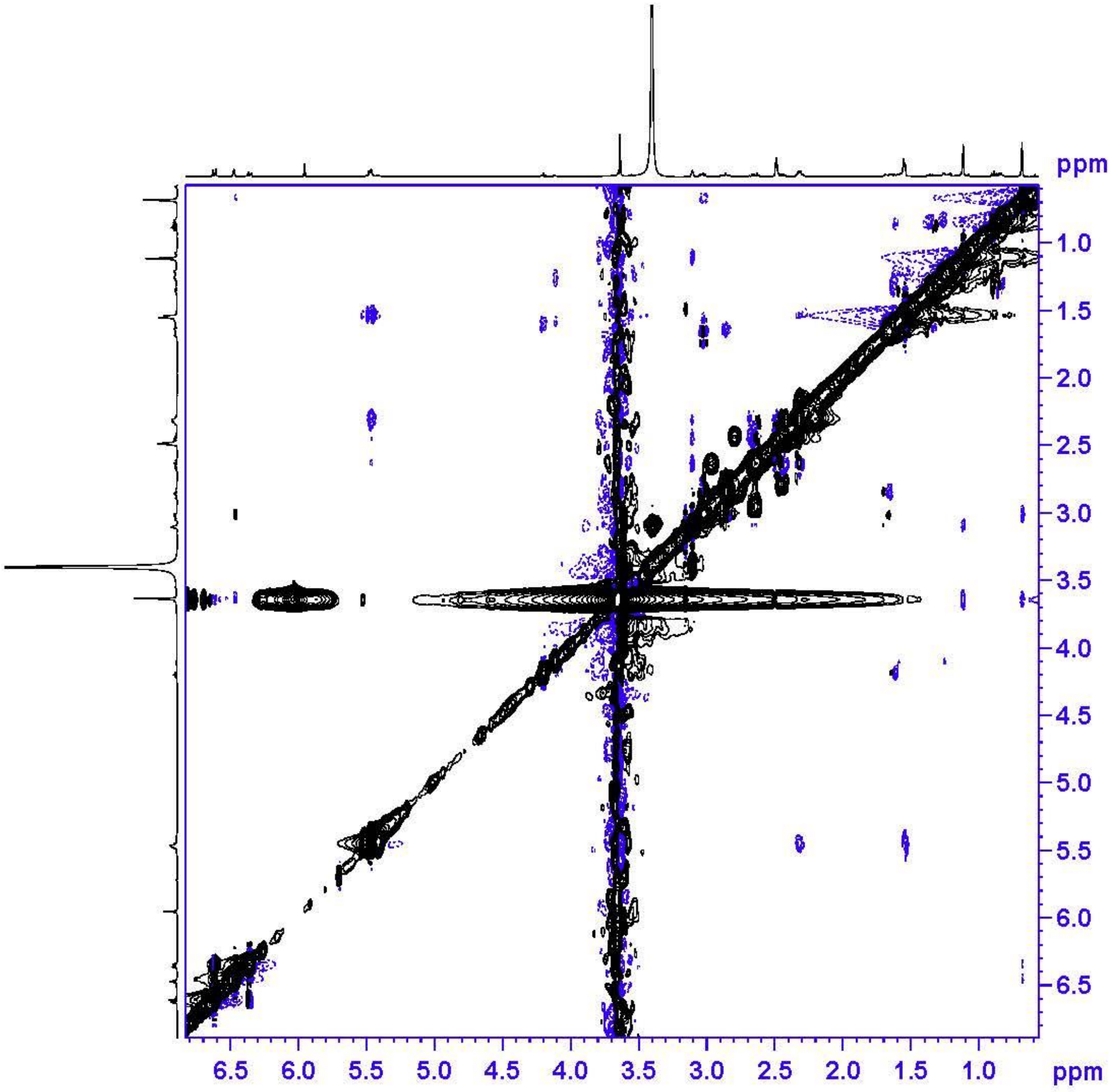

Current Data Parameters
NAME PA3-4 DMSO
EXPNO 7
PROCNO 1

F2 - Acquisition Parameters
Date_ 20191231
Time 15.17
INSTRUM spect
PROBHD 5 mm PABBO BB-
PULPROG noesygpphpp
TD 2048
SOLVENT DMSO
NS 94
DS 32
SWH 4401.409 Hz
FIDRES 2.149125 Hz
AQ 0.2326528 sec
RG 114
DW 113.600 usec
DE 6.50 usec
TE 292.3 K
D0 0.00009590 sec
D1 2.00000000 sec
D8 0.30000001 sec
D11 0.03000000 sec
D12 0.00002000 sec
D16 0.00020000 sec
IN0 0.00022720 sec

======== CHANNEL f1 ========
SFO1 400.1322007 MHz
NUC1 1H
P1 13.90 usec
P2 27.80 usec
P17 2500.00 usec
PLW1 12.14299965 W
PLW10 3.47059989 W

====== GRADIENT CHANNEL =====
GPNAM[1] SMSQ10.100
GPZ1 40.00 %
P16 1000.00 usec

F1 - Acquisition parameters
TD 256
SFO1 400.1322 MHz
FIDRES 17.193003 Hz
SW 11.000 ppm
FnMODE States-TPPI

F2 - Processing parameters
SI 1024
SF 400.1300013 MHz
WDW QSINE
SSB 2
LB 0 Hz
GB 0
PC 1.00

F1 - Processing parameters
SI 1024
MC2 States-TPPI
SF 400.1300052 MHz
WDW QSINE
SSB 2
LB 0 Hz
GB 0

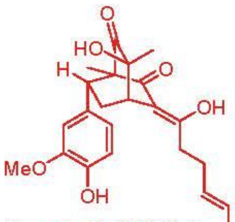

Recorded in DMSO-d_6

Supplementary Figure S12. NOESY spectrum of compound **2**
